# Current Understanding of the Acute Exacerbation of Chronic Rhinosinusitis

**DOI:** 10.3389/fcimb.2019.00415

**Published:** 2019-12-04

**Authors:** Dawei Wu, Benjamin Saul Bleier, Yongxiang Wei

**Affiliations:** ^1^Department of Otolaryngology-Head and Neck Surgery, Beijing Anzhen Hospital, Capital Medical University, Beijing, China; ^2^Department of Otolaryngology, Massachusetts Eye and Ear Infirmary, Harvard Medical School, Boston, MA, United States

**Keywords:** Chronic rhinosinusitis, acute exacerbations, bacteria, virus, etiology, treatment

## Abstract

**Background:** Acute exacerbations of chronic rhinosinusitis (CRS) have been increasingly recognized as an important disease entity with a significant impact on the quality of life. There is a growing amount of research on the etiopathogenesis and management of acute exacerbations of CRS. This review aims to summarize the current literature and provide an overall understanding of acute CRS exacerbations.

**Methods:** A related literature review with the key terms of “chronic rhinosinusitis” and “exacerbation” was performed using PubMed.

**Results:** There is no consensus definition of the acute exacerbation of CRS. Impaired mucociliary clearance, atrophic rhinitis, and immunologic changes are important predisposing factors for acute CRS exacerbations. Current evidence supports the role of the transient viral infection as the initial inflammatory stimulus in the pathogenesis of acute CRS exacerbations. Secondary bacterial infection or microbial community dysbiosis within the sinonasal cavity is the main event during the acute exacerbation of CRS. Distinct changes in local and systemic immune responses during exacerbation provide new insights into the pathophysiology of CRS exacerbation. Although current guidelines suggest the use of short-term antibiotics in patients with acute CRS exacerbation for symptomatic relief, evidence-based treatment recommendations for acute CRS exacerbation are still lacking, and large-high-quality RCTs are required.

**Conclusion:** There have been significant advances in understanding the etiology and immunological feathers of acute CRS exacerbation. Nevertheless, consensus definition, diagnostic criterion, biomarkers to differentiate acute CRS exacerbation from CRS, assessment of disease severity, and evidence-based treatment options for acute CRS exacerbation are still lacking.

## Introduction

Chronic rhinosinusitis (CRS) is a highly heterogeneous upper airway disease (Orlandi et al., 2016), which affects about 11–12% of adults in western countries (Hastan et al., 2011; Hirsch et al., 2017) and ~8% of the general population in China (Shi et al., 2015). It significantly impairs quality of life due to the chronic symptoms (Hoehle et al., 2016) and acute exacerbations of CRS (Phillips et al., 2017). Notably, one significant issue related to the care of CRS patients is concerning the acute exacerbations, which might account for increasing healthcare cost (Chung et al., 2014; Smith et al., 2015) and annual physician visits (Smith et al., 2013), as well as significant decreases in workplace productivity (Rudmik et al., 2014; Campbell et al., 2017). An acute exacerbation of CRS is defined as an acute and transient worsening of preexisting symptoms in patients with CRS (Fokkens et al., 2012), and the frequency of CRS exacerbations is recently identified as an independent predictor of quality of life (Phillips et al., 2017). Currently, triggers leading to CRS disease exacerbation are not well-characterized. However, patients with acute CRS exacerbation are mainly attributed to the bacterial infection, and therefore they are treated as acute rhinosinusitis with antibiotics in the leading guidelines (Fokkens et al., 2012; Peters et al., 2014; Banoub et al., 2018).

There are accumulating evidence supporting the critical role of sinus mucosal microbiome in the pathogenesis of CRS (Hamilos, 2014; Brook, 2016; Mahdavinia et al., 2016; Wagner et al., 2017; Sivasubramaniam and Douglas, 2018), either as a direct driver of chronic inflammation (Hamilos, 2015; Lan et al., 2018; Lee et al., 2018) or as being potentially involved in its exacerbation (Wood et al., 2011; Tan et al., 2017). The etiology of acute CRS exacerbation is usually a secondary bacterial infection that commonly begins with a viral upper respiratory tract infection (Cho et al., 2013; Rowan et al., 2015) or begins with microbial community dysbiosis within the sinonasal cavity (Brook et al., 2005; Brook, 2006). Furthermore, the acute exacerbations of CRS after endoscopic surgery are highly associated with bacterial infections (Bhattacharyya and Kepnes, 1999; Bhattacharyya et al., 2004; Ikeda et al., 2011). Although viral and bacterial infections are the main event during the process of acute exacerbation, factors influencing the dynamics of the nasal microbiota, pathogenic mechanisms exerted by microbial dysbiosis, and the association between the nasal microbiota and outer stimuli remain unclear. Recent studies have proved that patients with acute CRS exacerbation have distinct immunological changes (Rank et al., 2013; Divekar et al., 2015), and a series of risk factors for the acute exacerbations of CRS have been identified (Kuiper et al., 2018), which promotes the understanding of the complex pathogenesis of acute CRS exacerbation. This review will summarize the current knowledge on clinical and immunologic characteristics and medical treatment of acute CRS exacerbation.

## Definition of Acute Exacerbation of Chronic Rhinosinusitis

There is no consensus definition of an acute exacerbation of CRS mainly due to the inconsistency in reporting of endpoints and the complex etiology of acute exacerbation. The International Consensus Statement on Allergy and Rhinology put forward the definition of AECRS that a sudden worsening of symptoms in a patient previously diagnosed with CRS, with a return to baseline symptoms after treatment (Orlandi et al., 2016). Similarly, the European Position Paper on Rhinosinusitis and Nasal Polyps 2012 (EPOS 2012) defined the AECRS based on the sudden worsening of preexisting sinonasal symptoms in patients with CRS (Fokkens et al., 2012). Furthermore, several empirical definition criteria have been widely used for research purposes. A study from Rank and colleagues defined a CRS exacerbation based on diagnosis coding and at least one of the following: a prescription for systemic antibiotics, systemic corticosteroids, plans for surgical intervention, emergency department or urgent care visit, or hospitalization for CRS (Rank et al., 2010). Similarly, Sedaghat and colleagues used three described metrics to assess the exacerbation frequency of CRS, the number of patient-reported (1) sinus infections, (2) CRS-related antibiotic courses, and (3) CRS-related oral corticosteroid courses, each in the last 3 months (Phillips et al., 2017; Banoub et al., 2018; Yamasaki et al., 2018). These direct treatment-related metrics of acute CRS exacerbation facilitate the quantitative research and assessment on the acute exacerbation of CRS, although there is a certain discrepancy between these metrics. It is necessary to put forward a well-recognized diagnostic criterion that contains either symptom-based or actual examination-based items.

It is also important to point out that recurrent acute rhinosinusitis (RARS), which shares a variety of symptoms with acute CRS exacerbation, is a distinct form of rhinosinusitis regarding anatomic variants (Sohn et al., 2018) and the number of previous sinus surgery (Poetker et al., 2008). RARS is defined by a threshold of 4 episodes of acute bacterial rhinosinusitis per year (Poetker et al., 2008; Bhattacharyya et al., 2012; Rosenfeld et al., 2015), and patients with RARS experience complete resolution of sinus-specific symptoms and have completed resolved sinus changes between episodes of acute bacterial rhinosinusitis (Barham et al., 2017). Furthermore, an appropriateness criterion for endoscopic sinus surgery in the management of adult RARS has been recently proposed to optimize the quality and value of surgical inventions (Rudmik et al., 2018).

## Predisposing Factors for Acute CRS Exacerbations

Previous studies have focused on the mechanisms of CRS (Bachert et al., 2018; Cao et al., 2018), and the etiology of acute CRS exacerbation is not well-identified (Orlandi et al., 2016). There has been an increasing consensus that predisposing conditions including allergic rhinitis, nasal deformity, immune deficiency, and other environmental factors create an environment conducive to the viral infection (Tan et al., 2017) and the subsequent growth of bacteria (Brook, 2006; Ikeda et al., 2011; Bose et al., 2016), leading to acute exacerbations. CRS is characterized by a dysfunctional host-environmental interaction both in the epithelium and the subsequent immune response (Tieu et al., 2009; Fokkens et al., 2012; Schleimer, 2017). Therefore, there are some certain defects of host immune response associated with CRS predispose to acute CRS exacerbation when the triggers are involved. It has been reported that impaired mucociliary clearance and atrophic rhinitis in combination with CRS are important predisposing factors for acute CRS exacerbations (Hafner et al., 1997; Fokkens et al., 2012; Dutta and Ghatak, 2013).

## Initiating Role of the Virus in the Acute CRS Exacerbation

Similar to the initiating role of the virus in the exacerbation of acute rhinosinusitis (Eloy et al., 2011; Fokkens et al., 2012), there is evidence supporting the crucial role of the virus in the acute CRS exacerbation and rhinovirus is the most prevalent virus in patients with CRS exacerbation (Cho et al., 2013; Rowan et al., 2015). A recent systematic review by Basharat et al. showed that the prevalence of rhinovirus infections is increased in patients with CRS. Furthermore, rhinovirus infections incite inflammatory reactions that may result in CRS exacerbations and progression of disease (Basharat et al., 2019). Human bocavirus and metapneumovirus (hMPV) are also found in the nasal washes of CRS during flare-ups (Tan et al., 2017). Moreover, some other viruses, such as herpes virus, human cytomegalovirus, and Epstein-Barr virus, have identified in patients with nasal polyps, and it is still unclear whether they contribute to the acute CRS exacerbations (Costa et al., 2014). Previous *in vitro* studies have shown that respiratory viral infection can facilitate the invasion of bacteria into the nasal mucosa through directly damaging to the epithelial cells and impairing tight junctions (Yeo and Jang, 2010; Wang et al., 2012). Furthermore, viral infections have been reported to obstruct sinus ostia (Gwaltney et al., 1994), production of inflammatory mediators by nasal epithelial cells (Wang et al., 2009), and damage to the cilia (Pedersen et al., 1983). It has been proposed that the priming of the nasal epithelium against acute viral infection potentiates its environment to be suitable for secondary bacterial infection, which may further exacerbate the symptoms (Tan et al., 2017).

It should also be noted that most acute viral infections in patients with CRS are self-limiting, and the duration of symptoms lasts <10 days (Orlandi et al., 2016). Interestingly, several studies have pointed out that viral colonization did not increase the viral infection rate in patients with CRS compared with controls (Liao et al., 2014), and the persistence of respiratory viruses within sinonasal mucosa is unlikely to be a cause of ongoing inflammation in CRS (Wood et al., 2011) but play an essential role in the symptom exacerbation in patients with CRS (Rowan et al., 2015). All this evidence supports the role of the transient viral infection as the initial inflammatory stimulus in the pathogenesis of acute CRS exacerbations. However, it is relatively harder to identify key viruses that exacerbate CRS compared to ARS due to the multifactorial nature of CRS exacerbation.

## Bacteriology in the Acute CRS Exacerbation

While bacteria may trigger acute infectious exacerbations (Bose et al., 2016), the role of bacteria in acute CRS exacerbation is not completely understood. The most commonly cultured microbiota during acute exacerbations of CRS included *Staphylococcus aureus, Pseudomonas aeruginosa*, and other common pathogens (*Streptococcus pneumonia, Haemophilus influenza, Moraxella catarrhalis, or Streptococcus pyogenes*) (Cincik and Ferguson, 2006; Liu et al., 2013). Besides, *S. pneumoniae* and *H. influenzae* were found more frequently in patients with acute CRS exacerbation compared to those with CRS without frequent acute exacerbations (Brook, 2006). Also, the identified organisms were predominantly anaerobic, which was similar to those generally identified in CRS (*Prevotella, Porhyromonas, Peptostreptococcus, and Fusobacterium subspecies*). A study by Merkley et al. concluded that bacterial abundance was increased, but diversity was decreased during acute exacerbations of CRS (Merkley et al., 2015). These studies support the hypothesis of microbial imbalance as a critical driver of acute CRS exacerbation.

As a chronic inflammatory airway disease, CRS is characterized by mucosal microbiota dysbiosis (Lee et al., 2018). Under a specific predisposing condition, patients with CRS experience acute and transient alternations of the mucosal microbiota, which may contribute to the sinus infection. It has been well-recognized that viral infection plays an initiating role in the pathogenesis of acute CRS exacerbation, and a bacterial infection occurs as the secondary event. It can be speculated that the host response to viral infection is likely to influence susceptibility to acute CRS exacerbation. A better understanding of the biologic mechanisms of host susceptibility to viral infection will be crucial for developing more effective preventions and treatments aimed at improving the quality of life and thereby reducing the high cost in patients with CRS.

## Immunological Feathers of acute Exacerbation of CRS

Acute exacerbation of CRS is increasingly being recognized as a distinct clinical manifestation of CRS that should be routinely evaluated in patients with CRS (Divekar et al., 2015; Banoub et al., 2018). Immunologic changes that occur during exacerbation may help physicians understand the pathogenesis of this acute phase of CRS. Rank and colleagues found that the levels of IL-6, eosinophil major basic protein (MBP), myeloperoxidase (MPO), eosinophil-derived neurotoxin, and uric acid in nasal secretion samples during CRSwNP exacerbation were significantly elevated when compared with the baseline measurements (Rank et al., 2013). What is more, the level of IL-6 significantly and positively correlated with both the levels of MBP and MPO. Increased IL-6 response has been associated with the onset of the viral upper respiratory tract infection in healthy individuals or virus-induced asthma exacerbations (Zhu et al., 1996; Jackson and Johnston, 2010). An epidemiological study also indicated that acute exacerbations of chronic rhinosinusitis occurred in the winter season during which viral infections are known to be prevalent, which implied a potential relationship between CRS disease activity and viral infection (Rank et al., 2010).

Systemic immune responses during exacerbation in patients with CRSwNP were also explored (Divekar et al., 2015). It showed that vascular endothelial growth factor (VEGF) and granulocyte–macrophage colony-stimulating factor (GM-CSF) levels in serum were significantly increased in CRSwNP patients during exacerbation when compared to control subjects or their baseline values. As the fact that VEGF and GM-CSF may play essential roles in tissue remodeling, and innate and adaptive immunity of nasal and sinus mucosa, future studies are warranted to investigate their exact role in the pathological changes in patients with CRS during exacerbation. These works have provided new insights into the pathophysiology of CRSwNP exacerbation. However, it should also be pointed out that the animal model of CRS with acute exacerbation is still lacking, and a new model would be helpful to elucidate its pathophysiological mechanism.

## Current Medical Treatment for Patients with Acute CRS Exacerbation

Current guidelines recommend the use of short-term antibiotics in patients with acute CRS exacerbation for symptomatic relief (Fokkens et al., 2012; Peters et al., 2014; Orlandi et al., 2016). Non-macrolide antibiotics (i.e., cefuroxime, ofloxacin, cefixime, amoxicillin with or without clavulanic acid) comprises the mainstay of the short-term antibacterial therapy in CRS patients with the most significant benefit in CRS exacerbations with a positive culture (Gehanno and Cohen, 1993; Huck et al., 1993; Namyslowski et al., 2002; Adelson and Adappa, 2013; Hoza et al., 2013). There is only one RCT study about the antibiotics treatment of acute CRS exacerbation (Sabino et al., 2017). It showed that amoxicillin-clavulanate for 14 days did not change the clinical course of acute CRS exacerbation compared with placebo and the addition of an oral antibiotic to ongoing topical intranasal steroid spray did not provide additional benefit during the management of acute CRS exacerbation. The study also pointed out that the sample size (*n* = 21) was relatively small, which might lead to the inherent risks of committing biased conclusions. A recent review has discussed the role of macrolide treatment in patients with acute CRS exacerbation (Oakley et al., 2017). The high-dose macrolide antibiotics are typically involved in the treatment of acute CRS exacerbation, especially in penicillin-allergic patients, and a therapeutic dose is usually administered for 10 days. However, the current literature about the macrolide therapy that focused on the acute CRS exacerbation is still lacking, and further studies are needed in the future. In addition to antibiotic treatment, other medical treatment has also been reported. A retrospective study by Chaudhry et al. showed that topical triamcinolone acetonide (TA) and carboxymethylcellulose (CMC) foam for acute exacerbation of CRSwNP could significantly decrease SNOT-22 scores and the overall prednisone use in postoperative CRSwNP patients (Chaudhry et al., 2014). A recent review by Miyake and Bleier pointed out that topical medications might have an advantage over oral antibiotics for the treatment of acute CRS exacerbations (Miyake and Bleier, 2019). Lacking evidence-based treatment recommendations for acute CRS exacerbation, therefore, requires further studies on this disease status, and large-high-quality RCTs would help determine the best medical therapy to manage patients with acute CRS exacerbation.

## Conclusion and Future Research Direction

Acute CRS exacerbation has gained increasing attention due to its significant influence on patients' quality of life and healthcare cost. As a distinct form of CRS, acute CRS exacerbation can be triggered by viruses and then follows with bacterial infections, which might be the result caused by the imbalance among the bacterial species within the sinus cavities. There have been significant advances in understanding the etiology and immunological feathers of acute CRS exacerbation. Nevertheless, consensus definition, diagnostic criterion, biomarkers to differentiate acute CRS exacerbation from CRS, assessment of disease severity, and evidence-based treatment options for acute CRS exacerbation are still lacking.

## Author Contributions

DW drafted the manuscript. BB and YW reviewed and revised this manuscript.

### Conflict of Interest

The authors declare that the research was conducted in the absence of any commercial or financial relationships that could be construed as a potential conflict of interest.
